# Key indicators for guiding tocilizumab therapy to prevent orbital decompression surgery in hormone-resistant dysthyroid optic neuropathy

**DOI:** 10.3389/fimmu.2025.1556742

**Published:** 2025-05-29

**Authors:** Wenjun Shu, Mingxu Yan, Lu Gan, Lu Li, Hongyue Tao, Zhiyu Peng, Jinghan Wang, Xiaofeng Li, Xintong Lin, Haifeng Chen, Jie Guo, Kang Xue, Hongguang Cui, Heng Lou, Binbin Xu, Jinwei Cheng, Hongying Ye, Yiming Li, Jiang Qian, Rui Zhang, Ruiqi Ma, Fangfang Zeng

**Affiliations:** ^1^ Eye Institute and Department of Ophthalmology, Eye & ENT Hospital, Fudan University, Shanghai, China; ^2^ NHC Key Laboratory of Myopia and Related Eye Diseases; Key Laboratory of Myopia and Related Eye Diseases, Chinese Academy of Medical Sciences, Shanghai, China; ^3^ Shanghai Key Laboratory of Visual Impairment and Restoration, Eye & ENT Hospital, Fudan University, Shanghai, China; ^4^ School of Basic Medical Sciences, Fudan University, Shanghai, China; ^5^ Department of Endocrinology and Metabolism, Huashan Hospital, Fudan University, Shanghai, China; ^6^ Department of Radiology and Institute of Medical Functional and Molecular Imaging, Huashan Hospital, Fudan University, Shanghai, China; ^7^ Department of Ophthalmology, The First Affiliated Hospital, Zhejiang University School of Medicine, Hangzhou, Zhejiang, China

**Keywords:** magnetic resonance imaging, optic neuropathy, thyroid-associated ophthalmopathy, thyroid-stimulating hormone receptor antibody, tocilizumab

## Abstract

**Introduction:**

Tocilizumab (TCZ) has been demonstrated to be effective in treating thyroid-associated ophthalmopathy (TAO); however, its efficacy in hormone-resistant dysthyroid optic neuropathy (DON) remains unclear. This study aims to identify baseline and post-treatment indicators that can predict the necessity for surgical intervention following TCZ therapy.

**Methods:**

Thirty-one hormone-resistant DON patients treated with TCZ were categorized into surgery (n=15, 7 males, 8 females) and non-surgery (n=16, 8 males, 8 females) groups based on their post-TCZ surgical status. Retrospective comparisons between the two groups were performed using pre- and post-treatment ophthalmic assessments, biomarkers, and orbital magnetic resonance imaging (MRI) scans. The predictive value of identified variables was evaluated through receiver operating characteristic (ROC) curve analysis and logistic regression analysis.

**Results:**

The surgery group exhibited a significantly higher baseline thyroid-stimulating hormone receptor antibody (TRAb) (*P*=0.001) and a positive change in the maximal signal intensity ratio of extraocular muscle to temporalis muscle (SIR(EOM/temporalis)_MAX_), whereas the non-surgery group demonstrated a negative change (*P*<0.001). A TRAb cut-off value of ≤5.07 IU/L predicted non-surgery with 93.3% sensitivity and 81.2% specificity, while a SIR(EOM/temporalis)_MAX_ cut-off value of ≤-1.83 had 86.7% sensitivity and 87.5% specificity. The area under the curve was 0.846 for TRAb and 0.863 for SIR(EOM/temporalis)_MAX_. Multivariate regression confirmed SIR(EOM/temporalis)_MAX_ change (*P*=0.017) as an independent predictor of surgical intervention. Linear regression revealed a significant correlation between SIR(EOM/temporalis)_MAX_ change and TCZ dosage in the non-surgery group (*P*=0.005), but not in the surgery group.

**Discussion:**

The percentage change in SIR(EOM/temporalis)_MAX_ following TCZ treatment and baseline TRAb can serve as predictors of the necessity for surgical intervention in hormone-resistant DON. These indicators assist in the personalization of TCZ therapy and in the avoidance of unnecessary surgical procedures.

## 1 Introduction

Thyroid-associated ophthalmopathy (TAO) represents the most common extraocular manifestation of Graves’ disease (GD) (1), with approximately 30-35% of individuals with GD develop TAO. While its pathophysiological mechanisms remain incompletely understood, the precise etiology of GD has also not been identified. The female-to-male ratio for TAO is 3-4:1. A smaller subset, approximately 5%, progresses to dysthyroid optic neuropathy (DON) (2). DON is characterized by visual impairment resulting from TAO after excluding other potential causes of visual dysfunction (3). In the absence of treatment, DON can lead to irreversible vision loss (4). The diagnosis of DON typically involves a combination of clinical assessments, such as evaluation of visual acuity, color vision, and central critical fusion frequency, alongside radiological imaging. Magnetic resonance imaging (MRI) is particularly effective for detecting extraocular changes, such as swelling and inflammation of the extraocular muscles and/or orbital adipose tissue, using T2-weighted coronal images with fat suppression(FS) with short T1 inversion recovery (STIR) sequences. These imaging findings are essential for assessing disease activity and planning surgical intervention.

Standard treatment options for DON include intravenous corticosteroids (IvCGs) (5). However, in cases where steroid therapy is ineffective, urgent orbital decompression may be necessary within a two-week timeframe. The acute phase of DON, often accompanied by unstable thyroid function, introduces additional risks, necessitating meticulous management of general anesthesia and addressing potential intraoperative and postoperative complications. Patients with TAO frequently exhibit elevated serum levels of interleukin-6 (IL-6) and its receptor (IL-6R), which activate immune cells and contribute to tissue proliferation. This activation results in thickening of the extraocular muscles, proptosis, and restricted ocular motility. Tocilizumab (TCZ), a recombinant human monoclonal antibody targeting IL-6R, inhibits IL-6 binding and attenuates inflammation, thereby potentially improving ocular symptoms.

Despite the benefits of TCZ, not all patients with TAO-induced DON experience significant clinical improvements; approximately 10%-20% fail to achieve substantial reductions in clinical activity score (CAS) (6, 7). As a result, surgical intervention may still be necessary for certain patients. A notable gap remains in systematic clinical research regarding which patients may avoid surgery following TCZ treatment. This study seeks to address this gap by analyzing changes in MRI and laboratory data from patients with hormone-resistant DON due to TAO before and after TCZ treatment. The objective is to identify indicators that could inform surgical decision-making and facilitate more personalized treatment strategies for patients with DON.

## 2 Methods

### 2.1 Patients

This study received approval from our institutional ethical review board and involved a retrospective analysis of de-identified patient data. Informed consent was obtained from all participants, and their personal information was anonymized to ensure privacy and adherence to ethical standards.

A total of 31 patients (mean age 49.19 ± 6.95 years; 15 males, 16 females) diagnosed with TAO and hormone-resistant DON were enrolled between January 2023 and September 2024. These patients represented all those diagnosed with these conditions within this timeframe who met the following inclusion criteria: age between 18 and 65 years, TAO symptoms present for ≤12 months, thyroid function within normal or near-normal ranges, no prior ocular surgery in the affected eye. Diagnosis of DON was based on acute visual loss, with or without painful eye movement, and the presence of at least two of the following criteria: relative afferent defect, visual field defects, visual evoked potential abnormalities, and color vision deficits. Alternative diagnoses, including ischemic, traumatic, compressive, infiltrative, toxic, or hereditary optic neuropathies, were excluded. Resistance to hormone therapy was defined as a decrease in the CAS of less than 2 following treatment, recurrence or persistence of TAO with an increase in CAS, development of DON during or after hormone therapy. Patients were categorized into surgery and non-surgery groups based on whether they underwent surgical intervention following TCZ treatment.

### 2.2 Data collection

Data collection encompassed ocular physical examinations (including intraocular pressure, proptosis, and palpebral fissure height), thyroid function tests, complete blood counts, biochemical indices, liver and kidney function tests, coagulation profiles, and cytokine levels. Orbital MRI was performed for each patient, focusing on the eye with the most severe TAO symptoms. IL-6 and thyroid-stimulating hormone receptor antibody (TRAb) levels were measured using the Elecsys assay.

### 2.3 MRI scanning and image analysis

MRI scanning was conducted using a 3.0 T MRI scanner (GE, USA) with the following imaging protocol: (1) axial T1-weighted and T2-weighted imaging with FS: field of view (FOV), 140 mm x 140 mm; slice thickness, 3.0 mm; slices, 15. (2) coronal T1-weighted imaging with FS and T2-weighted imaging with FS and STIR: FOV, 140 mm x 140 mm; slice thickness, 3.0 mm; slices, 15.

Two imaging specialists, blinded to clinical details, independently analyzed the images. Measurements included: (1) Extraocular muscle volume (MV): files were imported into 3D Slicer (version 5.6.2). Volume delineation was conducted using the “Segmentation Editor” and “Volumes” modules, with fine adjustments via the “Paint” and “Erase” tools. Three-dimensional volume data were exported from the “Models” interface. (2) Barrett’s index: calculated according to the methodology of Barrett et al. (8). (3) Signal intensity ratios (SIR): measured on T2-weighted coronal images with FS. The signal intensity of extraocular muscles and the ipsilateral temporalis muscle was measured using the region of interest (ROI) method. ROIs were drawn around the entire extraocular muscle and a 0.650 ± 0.050 cm² area of the ipsilateral temporalis muscle. Additionally, the ratio of the maximum lacrimal gland signal intensity to that of the ipsilateral temporalis muscle was determined using an ROI comprising 10–15% of the lacrimal gland cross-sectional area. (4) Optic nerve-to-white matter ratio: measured using the method described by Song et al. (1). (5) Axial length (AL): measured from the corneal apex to the anterior retinal surface on the largest ocular axial section. (6) Ocular medial fat thickness (OFT): measured on T1-weighted axial images as the maximum thickness of the fat from the lateral wall of the medial fat to the medial wall of the orbit at the level displaying the largest eye and optic nerve. (7) Lacrimal gland prominence (LGH): measured on T2-weighted axial FS images as the vertical distance from the anterior margin of the lacrimal gland to the anterior margin of the zygomatic arch at the clearest axial level.

### 2.4 Statistical analysis

Statistical analyses were performed using IBM SPSS software (version 20) and R Studio software (versions 4.3.1, 4.4.1, and 4.4.2). Continuous variables were reported as means ± standard deviations, with normality assessed via the Kolmogorov–Smirnov test. Depending on the data distribution, either parametric tests (independent samples t-test) or nonparametric tests (Mann–Whitney U test) were applied for between-group comparisons, and the Wilcoxon signed-rank test was used for paired pre- versus post-treatment comparisons. Categorical variables were analyzed using chi-square or Fisher’s exact tests, as appropriate. Receiver operating characteristic (ROC) curves were generated to evaluate predictive model performance, with area under the curve (AUC), optimal cut-off values, sensitivity, and specificity reported. Variables with *P <*0.10 in univariate logistic regression were entered into multivariate logistic regression models. Spearman or Pearson correlation tests (based on the normality) and linear regression analyses were performed to examine relationships between variables.

## 3 Results

### 3.1 Baseline clinical parameters

**Figure 1:**
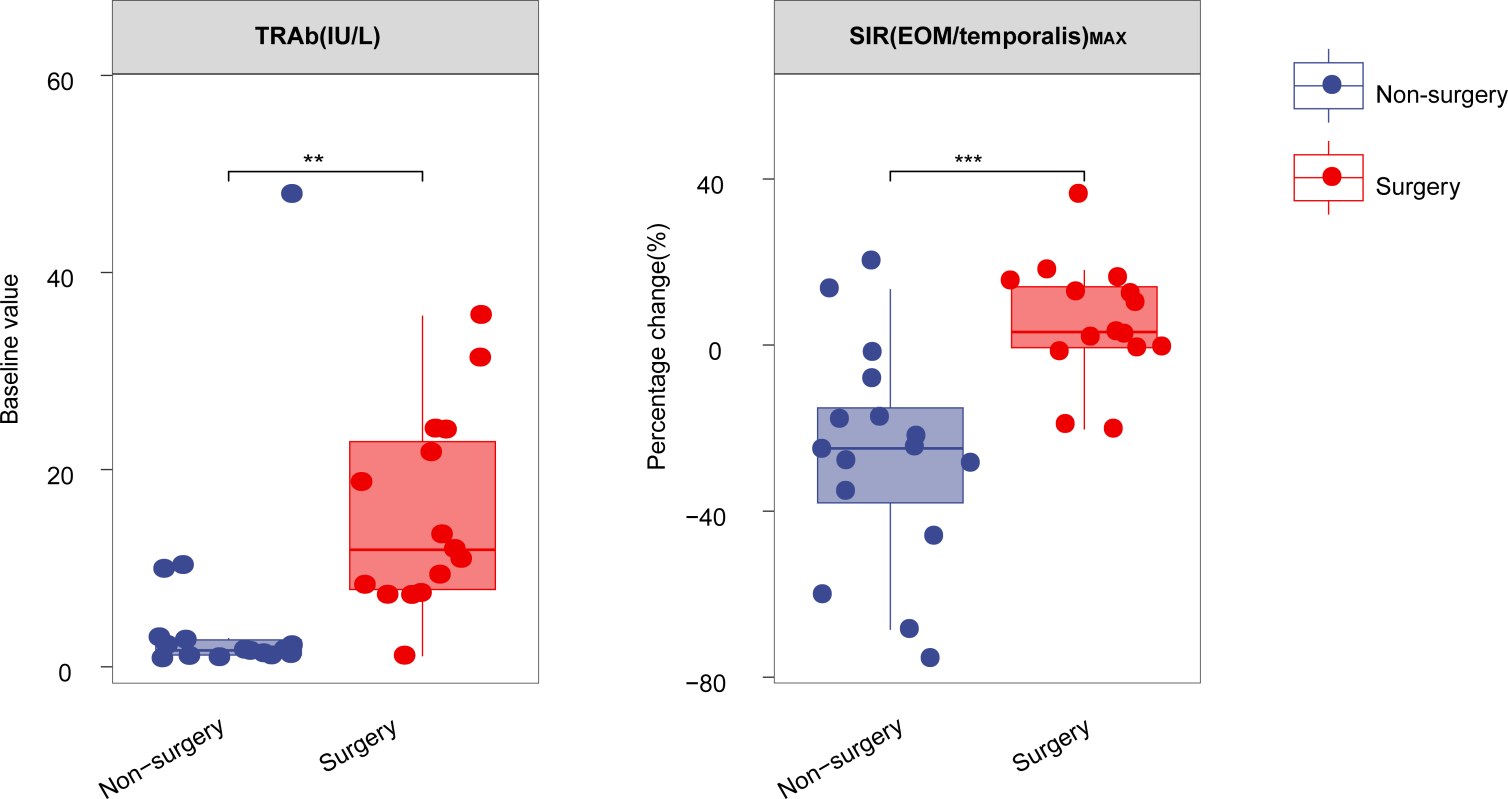
Boxplot comparing the percentage change in TRAb and SIR(EOM/temporalis)_MAX_ after one course of TCZ treatment between surgery and non-surgery groups. **P < 0.01, ***P < 0.001. All *P* values are two-sided. TRAb, thyroid-stimulating hormone receptor antibodies, SIR(EOM/temporalis)_MAX_: the maximal signal intensity ratio of extraocular muscle to temporalis muscle.

A total of 31 patients meeting the inclusion and exclusion criteria were diagnosed with DON. Fifteen patients were allocated to the surgery group and 16 to the non-surgery group. Baseline clinical parameters are detailed in **Table 1**, **Supplementary Tables 1, 2**. The surgery group exhibited significantly higher TRAb (*P*=0.001) (**Figure 1**, **Table 1**) compared with the non-surgery group. No significant differences were observed in baseline IL-6 (*P*=0.646), and cumulative hormone dosages did not differ significantly between the groups (*P*=0.493) (**Table 1**).

**Table 1:** Baseline data for laboratory and MRI parameters.

Parameters	Surgery	Non-surgery	*P* value*
Patients	15	16	
Male/female	7/8	8/8	1.000 ^a^
Age(years)	50.87 ± 6.60	47.63 ± 7.12	0.200 ^b^
Hormone(g)	3.41 ± 0.83	3.62 ± 0.80	0.493 ^b^
GO duration(d)	14.27 ± 2.12	13.62 ± 1.63	0.351 ^b^
GD duration(d)	381.13 ± 112.77	408.00 ± 133.33	0.551 ^b^
ATD(mg)	1482.43 ± 256.17	1556.38 ± 395.31	0.544 ^b^
TRAb(IU/L)	15.47 ± 9.96	5.57 ± 11.66	**0.001** ^c^
TSH(IU/L)	1.87 ± 1.73	2.44 ± 4.05	0.618 ^b^
FT3(pmo1/L)	4.64 ± 0.93	4.90 ± 0.86	0.429 ^b^
FT4(pmo1/L)	16.61 ± 2.93	17.09 ± 3.49	0.680 ^b^
IL-6(pg/ml)	2.17 ± 1.11	2.57 ± 2.65	0.646 ^c^
CAS	4.87 ± 1.30	4.75 ± 1.88	0.843 ^b^
Intraocular pressure(mmHg)	20.11 ± 4.21	18.63 ± 3.67	0.302 ^b^
Proptosis(mm)	19.87 ± 3.66	19.25 ± 2.82	0.602 ^b^
PFH(mm)	9.00 ± 2.00	8.63 ± 2.03	0.609 ^b^
MV(cm^3^)	7.27 ± 2.04	7.34 ± 1.49	0.914 ^b^
SIR(EOM/temporalis)_MAX_	3.50 ± 1.37	3.18 ± 1.01	0.455 ^b^
SIR(EOM/temporalis)_MIN_	2.01 ± 0.64	1.78 ± 0.70	0.236 ^c^

*GO*, Graves’ Orbitopathy; *GD*, Graves’ Disease*; ATD*, antithyroid drug*; TRAb*, thyroid-stimulating hormone receptor antibodies; *TSH*, thyroid-stimulating hormone; *FT3*, free triiodothyronine; *FT4*, free thyroxine; *IL-6*, interleukin-6; *CAS*, clinical activity score; *PFH*, palpebral fissure height; *MV*, muscle volume; *SIR(EOM/temporalis)_MAX_*, the maximal signal intensity ratio of extraocular muscle to temporalis muscle; *SIR(EOM/temporalis)_MIN_*, the minimal signal intensity ratio of extraocular muscle to temporalis muscle.

a Chi-square test.

b Independent sample t-test.

c Mann–Whitney U test.

*P < 0.05 are marked in bold.

### 3.2 Changes of clinical parameters after TCZ treatment

**Figure 2:**
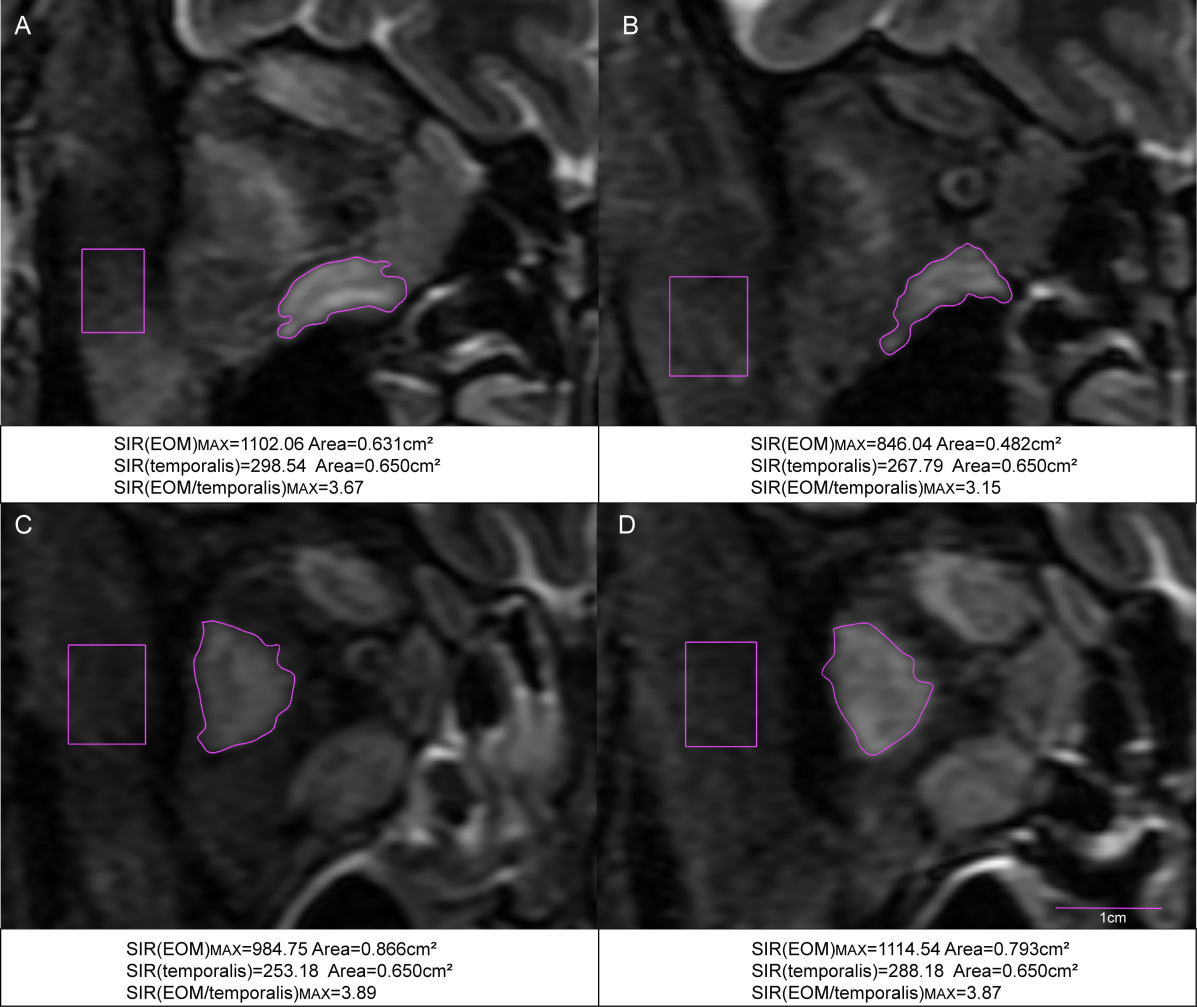
Coronal STIR MRI scans of the extraocular muscles in TAO patients with hormone-resistant DON. **(A)** Pre-treatment MRI of the right eye in a patient who did not require surgery. **(B)** Post-treatment MRI of the same patient after one course of TCZ treatment. **(C)** Pre-treatment MRI of the right eye in a patient who underwent surgery. **(D)** Post-treatment MRI of the same patient after one course of TCZ treatment. SIR(EOM/temporalis)_MAX_: the maximal signal intensity ratio of extraocular muscle to temporalis muscle.

Data on laboratory and imaging parameters were collected before and after one course of TCZ treatment. Analysis of the percentage changes revealed significant differences between the surgery and non-surgery groups in MV (*P*=0.036) and the maximal signal intensity ratio of extraocular muscle to temporalis muscle (SIR(EOM/temporalis)_MAX_; *P*<0.001) (**Figures 1**, **2A–D**, **Table 2**). Specifically, SIR(EOM/temporalis)_MAX_ showed positive changes in the surgery group and negative changes in the non-surgery group (**Figures 1**, **2A–D**, **Table 2**).

**Table 2:** Changes of laboratory and MRI parameters after one course of TCZ treatment.

Parameters	Surgery	Non-surgery	*P* value*
Patients	15	16	
Male/female	7/8	8/8	1.000 ^a^
Age(years)	50.87 ± 6.60	47.63 ± 7.12	0.200 ^b^
TRAb(IU/L)*	-31.48 ± 14.02	-31.81 ± 12.43	0.678 ^c^
MV(cm^3^)*	2.20 ± 14.38	-7.79 ± 15.50	**0.036** ^c^
SIR(EOM/temporalis)_MAX_*	5.75 ± 14.30	-26.61 ± 26.70	**<0.001** ^b^
SIR(EOM/temporalis)_MIN_*	1.80 ± 18.23	-0.44 ± 9.86	0.635 ^c^

*TRAb*, thyroid-stimulating hormone receptor antibodies; *MV*, muscle volume; *SIR(EOM/temporalis)_MAX_*, the maximal signal intensity ratio of extraocular muscle to temporalis muscle; *SIR(EOM/temporalis)_MIN_*, the minimal signal intensity ratio of extraocular muscle to temporalis muscle.

^a^Chi-square test.

Independent sample t-test.

^c^Mann–Whitney U test.

*TRAb(IU/L), MV(cm^3^), SIR(EOM/temporalis)_MAX_ and SIR(EOM/temporalis)_MIN_ refer to percentage changes observed after one course of TCZ treatment.

*P < 0.05 are marked in bold.

Conversely, no significant differences were found in TRAb levels (*P*=0.678) or the minimum signal intensity ratio of extraocular muscles to temporalis muscle (SIR(EOM/temporalis)_MIN_; *P*=0.635) between groups (**Table 2**). Following two courses of TCZ treatment, the percentage change in SIR(EOM/temporalis)_MAX_ remained significantly different between groups (*P*=0.034) (**Supplementary Table 3**). The surgery group exhibited a positive percentage change, whereas the non-surgery group showed a negative percentage change. This accentuated divergence suggests reduced efficacy of prolonged TCZ therapy in the surgery cohort.

Although TRAb levels declined significantly after one TCZ course (9.74 ± 10.55 vs 7.12 ± 8.37, Wilcoxon Signed-Rank Test, *P*<0.001), IL-6 levels exhibited a non-significant upward trend (2.40 ± 2.02 vs 4.00 ± 7.06, Wilcoxon Signed-Rank Test, *P*=0.371). These results indicate that early changes in SIR(EOM/temporalis)_MAX_ may serve as a predictor of subsequent surgical necessity in hormone-resistant DON.

### 3.3 Identification of key indicators for guiding TCZ therapy to prevent surgery

**Figure 3:**
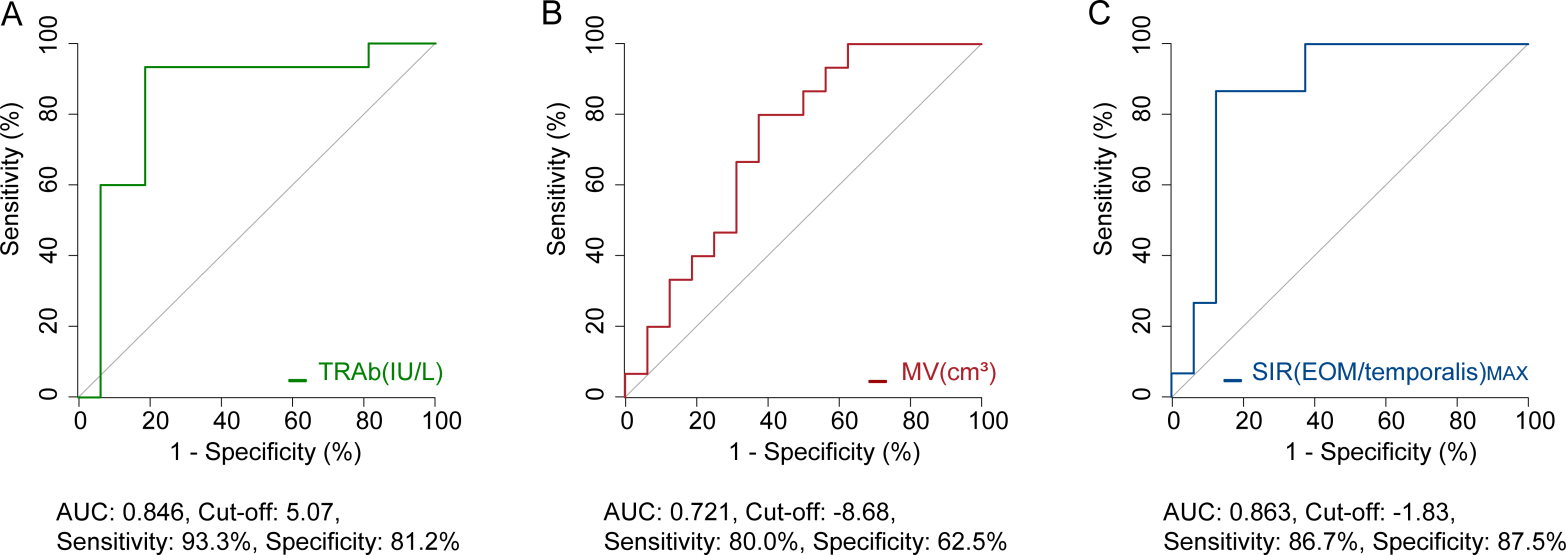
ROC analysis of potential indicators to prevent surgery. **(A)** Predictive value of baseline TRAb. **(B)** Predictive value of percentage change in MV following one course of TCZ treatment. **(C)** Predictive value of percentage change in SIR(EOM/temporalis)_MAX_ following one course of TCZ treatment. AUC, area under curve; TRAb, thyroid-stimulating hormone receptor antibodies; MV, muscle volume; SIR(EOM/temporalis)_MAX_, the maximal signal intensity ratio of extraocular muscle to temporalis muscle. Both MV and SIR(EOM/temporalis)_MAX_ refer to percentage changes observed after one course of TCZ treatment.

ROC analysis identified baseline TRAb (AUC=0.846) (**Figure 3A**), as well as post-treatment percentage changes in MV (AUC=0.721) and SIR(EOM/temporalis)_MAX_ (AUC=0.863) (**Figures 3B, C**), as significant predictors of whether TCZ could prevent the need for surgery. A baseline TRAb cut-off value of ≤5.07 IU/L predicted non-surgery with 93.3% sensitivity and 81.2% specificity (**Figure 3A**). A SIR(EOM/temporalis)_MAX_ cut-off value of ≤-1.83 showed 86.7% sensitivity and 87.5% specificity for non-surgery (**Figure 3C**).

**Figure 4:**
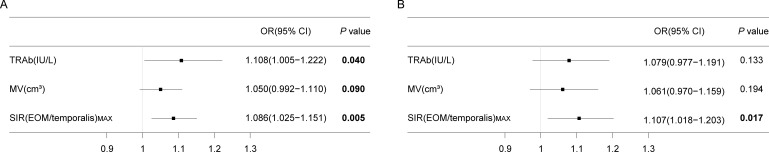
Logistic regression analysis results. **(A)** Univariate logistic regression results. **(B)** Multivariate logistic regression results. *P* < 0.10 are marked in bold. OR, odds ratio; CI, confidence interval; TRAb, thyroid-stimulating hormone receptor antibodies; MV, muscle volume; SIR(EOM/temporalis)_MAX_, the maximal signal intensity ratio of extraocular muscle to temporalis muscle. Both MV and SIR(EOM/temporalis) _MAX_ refer to the percentage changes observed after one course of TCZ treatment.

Univariate logistic regression analysis demonstrated that baseline TRAb, percentage change in MV, and SIR(EOM/temporalis)_MAX_ after one course of TCZ treatment were associated with the likelihood of requiring surgery (*P*<0.10) (**Figure 4A**). Multivariate logistic regression identified the percentage change in SIR(EOM/temporalis)_MAX_ (odds ratio=1.107, 95% confidence interval=1.018–1.203; *P*=0.017) as an independent predictor of surgical intervention (**Figure 4B**).

### 3.4 Correlation between key indicator changes and TCZ dosage

**Figure 5:**
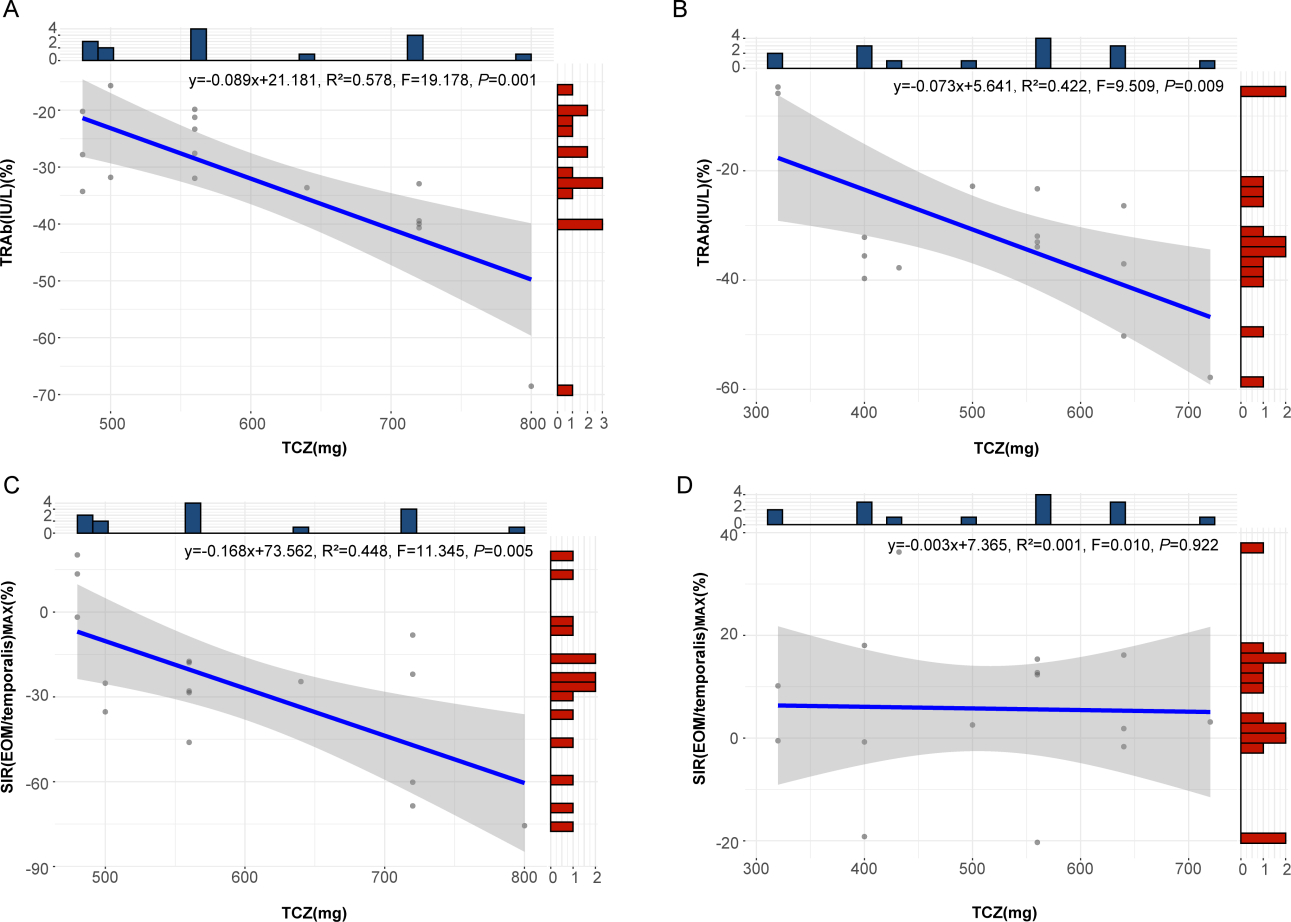
Regression curves for the percentage change in TRAb and SIR(EOM/temporalis)_MAX_ relative to TCZ dosage. **(A)** Regression curve showing the relationship between the percentage change in TRAb and TCZ dose in patients who did not undergo surgery. **(B)** Regression curve showing the relationship between the percentage change in TRAb and TCZ dose in patients who underwent surgery. **(C)** Regression curve showing the relationship between the percentage change in SIR(EOM/temporalis)_MAX_ and TCZ dose in patients who did not undergo surgery. **(D)** Regression curve showing the relationship between the percentage change in SIR(EOM/temporalis)_MAX_ and TCZ dose in patients who underwent surgery. TRAb, thyroid-stimulating hormone receptor antibodies; SIR(EOM/temporalis)_MAX_, the maximal signal intensity ratio of extraocular muscle to temporalis muscle; TCZ, tocilizumab. Both TRAb and SIR(EOM/temporalis) _MAX_ refer to the percent changes after TCZ treatment in the figure.

Correlation analysis revealed a significant association between the percentage change in TRAb after TCZ treatment and TCZ dosage in both the surgery group (*P*=0.009) and the non-surgery group (*P*=0.006). In contrast, percentage change in SIR(EOM/temporalis)_MAX_ were significantly correlated with TCZ dosage only in the non-surgery group (*P*=0.017), with no significant association in the surgery group (*P*=0.922) (**Supplementary Table 4**). Linear regression analysis confirmed these relationships for TRAb in both groups (**Figures 5A, B**) and for SIR(EOM/temporalis)_MAX_ in the non-surgery group (*P*=0.005) (**Figure 5C**), whereas no significant correlation was observed in the surgery group (*P*=0.922) (**Figure 5D**). These findings indicate that SIR(EOM/temporalis)_MAX_ changes more significantly with TCZ dosage in the non-surgery group, a trend not observed in the surgery group.

## 4 Discussion

This study highlights three primary findings regarding the management of TAO with hormone-resistant DON using TCZ. First, significant differences were observed between the surgery and non-surgery groups in the percentage change of SIR(EOM/temporalis)_MAX_ following one course of TCZ treatment, as well as in baseline TRAb. Second, both the percentage change in SIR(EOM/temporalis)_MAX_ after one course of TCZ treatment and baseline TRAb levels were independent predictors of the necessity for surgical intervention. Third, a correlation was observed in the non-surgery group between changes in SIR(EOM/temporalis)_MAX_ after TCZ treatment and the dosage administered, a trend absent in the surgery group. These findings suggest that monitoring changes in SIR(EOM/temporalis)_MAX_ post-TCZ treatment, along with baseline TRAb levels, may guide surgical decision-making in TAO patients with hormone-resistant DON.

Previous studies have utilized extraocular muscle SIR metrics to predict patient outcomes, with inconsistent results. Some investigations report that lower extraocular muscle SIR correlates with improved therapeutic response (9), whereas others find this relationship inconclusive (10). Our study evaluated the percentage change in SIR(EOM/temporalis)_MAX_ and SIR(EOM/temporalis)_MIN_ after one course of TCZ treatment. We found that changes in SIR(EOM/temporalis)_MAX_ significantly differed between the surgery and non-surgery groups and reliably predicted the need for surgery, whereas changes in SIR(EOM/temporalis)_MIN_ did not. This divergence may reflect the biphasic pathology of TAO, in which inflammation (11) and fibrosis (12, 13) coexist. T2-weighted MRI typically demonstrates elevated SIR in edematous regions and reduced SIR in fibrotic tissues. We therefore propose that SIR(EOM/temporalis)_MAX_ and SIR(EOM/temporalis)_MIN_ represent inflammatory edema and focal fibrosis, respectively. Consequently, TCZ effectively reduced inflammatory edema in the non-surgery group but had a limited effect on fibrosis. These findings are consistent with TCZ’s mechanism of IL-6–mediated inflammation inhibition and with literature indicating diminished efficacy in fibrotic stages (14, 15).

We also observed several novel findings in the laboratory analyses. Baseline TRAb levels were confirmed as a significant predictor, consistent with prior research (16, 17). Elevated baseline TRAb may prolong receptor activation in orbital fibroblasts, leading to increased orbital tissue volume and optic nerve compression (18). Since TCZ suppresses inflammation but does not eliminate existing TRAb, patients with high TRAb titers may respond suboptimally to TCZ and ultimately require surgical intervention. Although IL-6 promotes inflammation and fibrosis in ocular tissues (19, 20), no baseline difference in IL-6 levels was observed between surgery and non-surgery groups, indicating that IL-6 alone is not a reliable predictor of surgical necessity post-TCZ. The post-treatment rise in IL-6 may be a transient response due to the short interval between TCZ administration and IL-6 measurement, potentially influenced by TCZ’s binding to IL-6 receptors (21).

Limitations of this study include its retrospective design and small sample size, necessitating validation in larger cohorts. Constraints of current MRI technology may have affected measurement precision. Future investigations employing advanced imaging modalities, such as functional MRI, could yield more detailed microstructural insights and improve diagnostic accuracy. Additionally, certain parameters exhibited restricted value ranges due to assay limitations, potentially influencing quantitative outcomes.

In summary, the percentage change in SIR(EOM/temporalis)_MAX_ after one course of TCZ treatment appears to be a robust indicator for predicting the need for surgical intervention in hormone-resistant DON. MRI-based assessments of SIR(EOM/temporalis)_MAX_ and baseline TRAb levels offer valuable guidance for personalized, long-term management strategies in TAO patients with concomitant DON.

## Data Availability

The raw data supporting the conclusions of this article will be made available by the authors, without undue reservation.
