# Supplementary material for: Key indicators for guiding tocilizumab therapy to prevent orbital decompression surgery in hormone-resistant dysthyroid optic neuropathy
Source: Front Immunol. 2025 May 29;16:1556742. doi: 10.3389/fimmu.2025.1556742 (PMC12159062; doi:10.3389/fimmu.2025.1556742)
Supplement: Supplementary file 1 [file DataSheet1.pdf]

Supplementary Table 1 Additional baseline laboratory data for surgery and non-surgery groups

	Surgery	Non-surgery	*P* value*
Patients	15	16	
Male/female	7/8	8/8	1.000 [a]
Age(years)	50.87±6.60	47.63±7.12	0.200 [b]
Glucose(nmol/L)	6.79±1.90	6.33±0.98	0.782 [c]
RBC(x10^12/L)	4.80±0.38	4.97±0.30	0.185 [c]
Hemoglobin(g/L)	135.00±14.49	144.00±13.89	0.089 [b]
WBC(x10^9/L)	7.30±2.90	6.80±2.15	0.861 [c]
ANC(x10^9/L)	4.53±2.35	4.08±1.65	0.520 [c]
Neutrophils(%)	60.60±7.38	59.09±8.80	0.607 [b]
Lymphocytes(%)	28.15±5.28	26.97±7.81	0.623 [b]
Monocytes(%)	6.47±1.43	7.25±1.59	0.123 [c]
Eosinophils(%)	1.44±1.00	2.03±1.05	0.120 [b]
Hct(%)	42.62±2.98	43.33±2.30	0.468 [b]
MCV(fl)	90.09±5.30	91.48±3.51	0.286 [c]
MCH(pg)	29.22±2.11	29.39±1.22	0.787 [b]

RDW(%)	12.96±1.10	12.80±0.38	0.719 [c]
PLT(x10^9/L)	270.93±71.29	283.56±72.77	0.629 [b]
ALB(g/L)	42.94±7.27	42.80±3.07	0.481 [c]
K+(mmol/L)	3.79±0.25	3.83±0.19	0.508 [c]
Na+(mmol/L)	140.27±3.84	140.44±1.97	0.879 [b]
TBIL(umol/L)	9.22±5.84	8.67±2.75	0.874 [c]
UA(mmol/L)	0.29±0.09	0.29±0.08	0.607 [c]
Urea(mmol/L)	5.23±1.54	4.76±0.80	0.797 [c]
CREA(umol/L)	51.73±7.84	56.44±12.98	0.394 [c]
GGT(U/L)	20.00±9.58	20.81±6.96	0.488 [c]
Calcium(mmol/L)	2.30±0.08	2.29±0.09	0.677 [c]
ALT(U/L)	23.07±11.39	21.56±6.94	0.921 [c]
AST(U/L)	19.33±4.01	18.81±3.66	0.905 [c]
eGFR(ml/min)	106.20±8.81	104.00±8.87	0.495 [b]
TSH(mIU/L)	1.83±1.54	2.48±4.02	0.592 [c]
TPOAb(U/ml)	140.37±205.13	64.77±150.08	0.343 [c]
TgAb(nmol/L)	313.78±1026.65	268.18±995.22	0.859 [c]

T4(nmol/L)	102.36±24.00	107.97±16.95	0.797 [c]
T3(pmol/L)	1.97±0.37	1.83±0.49	0.363 [b]
FT4(pmol/L)	15.94±3.32	17.09±3.49	0.354 [b]
FT3(pg/ml)	5.17±1.19	5.84±1.61	0.202 [b]
TG(ng/ml)	13.40±13.84	18.74±18.09	0.590 [c]
IL-10(pg/ml)	1.82±1.51	1.56±1.83	0.289 [c]
IFN-γ(pg/ml)	2.23±1.30	3.55±3.77	0.485 [c]
IL-17(pg/ml)	2.23±1.11	1.94±0.98	0.379 [c]
IL-1β(pg/ml)	2.42±1.15	3.36±3.36	0.812 [c]

RBC, red blood cells; *WBC*, white blood cells; *ANC*, absolute neutrophil count; *Hct*, hematocrit; *MCV*, mean corpuscular volume; *MCH*, mean corpuscular hemoglobin; *RDW*, red cell distribution width; *PLT*, platelets; *ALB*, albumin; *TBIL*, total bilirubin; *UA*, uric acid; *CREA*, creatinine; *GGT*, gamma-glutamyl transferase; *ALT*, alanine aminotransferase; *AST*, aspartate aminotransferase; *eGFR*, estimated glomerular filtration rate; *TSH*, thyroid-stimulating hormone; *TPOAb*, thyroid peroxidase antibodies; *TgAb*, thyroglobulin antibodies; *T4*, thyroxine; *T3*, triiodothyronine; *FT4*, free thyroxine; *FT3*, free triiodothyronine; *TG*, thyroglobulin; *IL-10*, interleukin-10; *IFN-γ*, interferon-gamma; *IL-17*, interleukin-17; *IL-1β*, interleukin-1 beta

[a]Chi-square test

[b]Independent sample t-test

[c]Mann–Whitney U test

**P* < 0.05 are marked in bold

Supplementary Table 2 Additional baseline MRI data for surgery and non-surgery groups

	Surgery	Non-surgery	*P* value*
Patients	15	16	
Male/female	7/8	8/8	1.000 [a]
Age(years)	50.87±6.60	47.63±7.12	0.200 [b]
EOV(cm^3)	20.78±4.90	20.09±4.36	0.770 [c]
MVI(%)	37.13±15.01	37.22±6.30	0.983 [b]
SIR(LG/temporalis)	2.32±0.79	1.87±0.71	0.109 [b]
SIR1(optic nerve/white matter)	4.88±3.07	4.80±2.93	1.000 [c]
SIR2(optic nerve/white matter)	3.80±1.80	3.79±2.48	0.991 [b]
SIR3(optic nerve/white matter)	2.68±1.20	2.56±1.65	0.520 [c]
SIR(MR/temporalis)	2.37±0.78	2.40±0.76	0.928 [b]
SIR(LR/temporalis)	3.27±1.38	3.12±1.11	0.747 [b]
SIR(SR/temporalis)	2.37±1.15	2.57±1.02	0.418 [c]
SIR(IR/temporalis)	3.40±1.05	3.25±0.94	0.670 [b]

SIR(SO/temporalis)	2.08±0.84	1.79±0.60	0.477 [c]
Barrett index(%)	38.99±10.11	36.43±9.74	0.478 [b]
AL(cm)	21.21±1.55	21.65±1.73	0.466 [b]
OFT(cm)	7.59±1.26	7.29±1.37	0.514 [c]
LGH(cm)	14.76±2.23	14.23±1.31	0.105 [c]
LGH/OFT	1.99±0.41	2.04±0.55	0.489 [c]

EOV, eye orbit volume; *MVI*, muscle volume index; *SIR(LG/temporalis)*, the ratio of the highest signal intensity of the lacrimal gland to that of the ipsilateral temporal muscle; *SIR1(optic nerve/white matter)*, *SIR2(optic nerve/white matter)* and *SIR3(optic nerve/white matter)*, the highest signal intensity ratios of the optic nerve to the ipsilateral cerebral white matter were measured 3 mm, 6 mm and 9 mm behind the eye; *SIR(MR/temporalis)*, *SIR(LR/temporalis)*, *SIR(SR/temporalis)*, *SIR(IR/temporalis)* and *SIR(SO/temporalis)*, the ratio of the signal intensity of the extraocular muscles (medial, lateral, superior, inferior rectus and superior oblique) to that of the ipsilateral temporal muscle; *AL*, axial length; *OFT*, orbital fat thickness; *LGH*, lacrimal gland height

[a]Chi-square test

[b]Independent sample t-test

[c]Mann–Whitney U test

**P* < 0.05 are marked in bold

Supplementary Table 3 Changes of laboratory and MRI parameters after two courses of TCZ treatment

	Surgery	Non-surgery	*P* value*
Patients	15	16	
Male/female	7/8	8/8	1.000 [a]
Age(years)	50.87±6.60	47.63±7.12	0.200 [b]
$SIR(EOM/temporalis)_{MAX}$*	41.87±43.33	-47.13±21.51	**0.034** [c]

$SIR(EOM/temporalis)_{MAX}$, the maximal signal intensity ratio of extraocular muscle to temporalis muscle

[a]Chi-square test

[b]Independent sample t-test

[c]Mann–Whitney U test

*$SIR(EOM/temporalis)_{MAX}$ refer to percentage changes observed after two courses of TCZ treatment

**P* < 0.05 are marked in bold

Supplementary Table 4 Correlation between parameters and TCZ dosage

	Surgery		Non-surgery	
	Correlation coefficient	*P* value*	Correlation coefficient	*P* value*
TRAb(IU/L)*	-0.646	**0.009** [a]	-0.651	**0.006** [b]

SIR(EOM/temporalis)$_{MAX}$*	-0.028	0.922 [a]	-0.587	**0.017** [b]

TRAb, thyroid-stimulating hormone receptor antibodies; *SIR(EOM/temporalis)$_{MAX}$*, the maximal signal intensity ratio of extraocular muscle to temporalis muscle

[a]Pearson correlation test

[b]Spearman correlation test

*TRAb(IU/L) and SIR(EOM/temporalis)$_{MAX}$ refer to percentage changes observed after one course of TCZ treatment

*$P < 0.05$ are marked in bold
